# Protective Effects and Network Analysis of Ginsenoside Rb1 Against Cerebral Ischemia Injury: A Pharmacological Review

**DOI:** 10.3389/fphar.2021.604811

**Published:** 2021-07-02

**Authors:** Weijie Xie, Xinyue Wang, Tianbao Xiao, Yibo Cao, Yumei Wu, Dongsheng Yang, Song Zhang

**Affiliations:** ^1^ Shanghai Mental Health Centre, School of Medicine, Shanghai Jiao Tong University, Shanghai, China; ^2^ First Affiliated Hospital of Guizhou University of Traditional Chinese Medicine, Guiyang, China; ^3^ School of Pharmacy, Guizhou University of Traditional Chinese Medicine, Guiyang, China; ^4^ Department of Anesthesiology, Renji Hospital, School of Medicine, Shanghai Jiao Tong University, Shanghai, China

**Keywords:** ginsenoside Rb1, ischemia and reperfusion injury, ischemia stroke, anti-inflammatory, antioxidant, antiapoptosis

## Abstract

Ischemic stroke is a leading cause of death and disability worldwide. Currently, only a limited number of drugs are available for treating ischemic stroke. Hence, studies aiming to explore and develop other potential strategies and agents for preventing and treating ischemic stroke are urgently needed. Ginseng Rb1 (GRb1), a saponin from natural active ingredients derived from traditional Chinese medicine (TCM), exerts neuroprotective effects on the central nervous system (CNS). We conducted this review to explore and summarize the protective effects and mechanisms of GRb1 on cerebral ischemic injury, providing a valuable reference and insights for developing new agents to treat ischemic stroke. Our summarized results indicate that GRb1 exerts significant neuroprotective effects on cerebral ischemic injury both *in vivo* and *in vitro*, and these network actions and underlying mechanisms are mediated by antioxidant, anti-inflammatory, and antiapoptotic activities and involve the inhibition of excitotoxicity and Ca2^+^ influx, preservation of blood–brain barrier (BBB) integrity, and maintenance of energy metabolism. These findings indicate the potential of GRb1 as a candidate drug for treating ischemic stroke. Further studies, in particular clinical trials, will be important to confirm its therapeutic value in a clinical setting.

## Introduction

When the brain becomes blocked by a blood clot and blood is prevented from reaching the brain, an ischemic stroke occurs. Ischemic stroke, approximately accounting for 85% of all diagnosed strokes, has the characteristics of high morbidity, high mortality, high disability, and high recurrence rates and is mainly caused by cerebral ischemia and reperfusion (I/R) injury (CIRI) (Turley et al., 2005; Eltzschig and Eckle, 2011). I/R is a pathological condition characterized by an initial restriction of blood supply to an organ followed by the subsequent restoration of perfusion and concomitant reoxygenation (Turley et al., 2005; Eltzschig and Eckle, 2011), which is one of the leading causes of death worldwide (Turley et al., 2005; Woodruff et al., 2011; Feigin et al., 2014). I/R injury mainly includes ischemic stroke, acute kidney injury, and myocardial infarction. Due to the interrupted blood supply to the CNS, cerebral infarction causes ischemic stroke and other forms of CNS injury. As these mechanisms often involve complex combinations of necrosis, apoptosis, necroptosis, and autophagy, ischemic stroke–related pathogenesis is not totally clear (Turley et al., 2005; Eltzschig and Eckle, 2011). Based on accumulating evidence, once an ischemic stroke occurs, the insufficient blood supply directly stimulates neurons, causes the accumulation of glutamate, overactivates a plethora of downstream signaling pathways, and increases the intracellular calcium concentration, which triggers energy metabolism disorders (Bolaños et al., 2009), oxidative stress (Chamorro et al., 2016), Ca^2+^ overload, excitatory neurotransmitter release, the immune-mediated inflammatory response following acute ischemic stroke, and its related apoptosis and necrosis processes (Kamel and Iadecola, 2012; Chamorro et al., 2016), finally resulting in ischemic infarction of the brain. Hence, the main aim of acute stroke treatment is to salvage the ischemic penumbra or volume of hypoperfused, nonfunctional, yet still viable tissue surrounding the infarcted core.

Currently, tissue plasminogen activator (TPA) is regarded as the main effective pharmacological therapy and drug for ischemic stroke (Turley et al., 2005; Woodruff et al., 2011; Chamorro et al., 2016). Scholars have conducted extensive studies and developed some neuroprotective drugs and other interventions for treating ischemic injury (Tuttolomondo et al., 2011; Woodruff et al., 2011), ranging from pharmacologically blocking neurotransmitter receptors to intercepting cell death pathways, as well as the induction of hypothermia or hyperoxygenation. However, most of these studies failed to show the efficacy of any of these promising strategies. The current existing neuroprotective drugs remain limited and insufficient for the clinical treatment needs for ischemic stroke (Turley et al., 2005; Woodruff et al., 2011; Chamorro et al., 2016). Therefore, the development of novel therapeutic strategies and agents for preventing and treating ischemic stroke is urgently needed.

*Panax ginseng* C. A. Mey and *P. notoginseng* (Burk) F. H. Chen are commonly used as natural medicinal plants in TCM (Hui et al., 2010; Bao-Ying et al., 2014; Yang et al., 2014; Xie et al., 2018a; Xie et al., 2018b), the roots and medicinal ingredients of which have been in use for several hundred years. Pharmacological studies have shown that *P. notoginseng, P. ginseng*, and their extracts have many functions (Mancuso and Santangelo, 2017; Kim, 2018), such as anti-inflammatory activity (Allison and Ditor, 2014; Zheng et al., 2014; Zhang et al., 2015; Jeon and Kim, 2016), antioxidant activity, blood glucose regulation (Xie et al., 2016; Zhang et al., 2016), insulin resistance improvement (Zhang and Jiang, 2012; Zhai et al., 2018), inhibition of neuronal apoptosis (Li et al., 2014; Hou et al., 2017; Yang et al., 2017), and neuroprotection (Xie et al., 2018a; Xie et al., 2018b; Wang et al., 2016; Berge and Riise, 2015). Hence, one of the main tasks is to identify natural active substances and compounds that can be utilized for the prevention and treatment of ischemic stroke.

**FIGURE 1:**
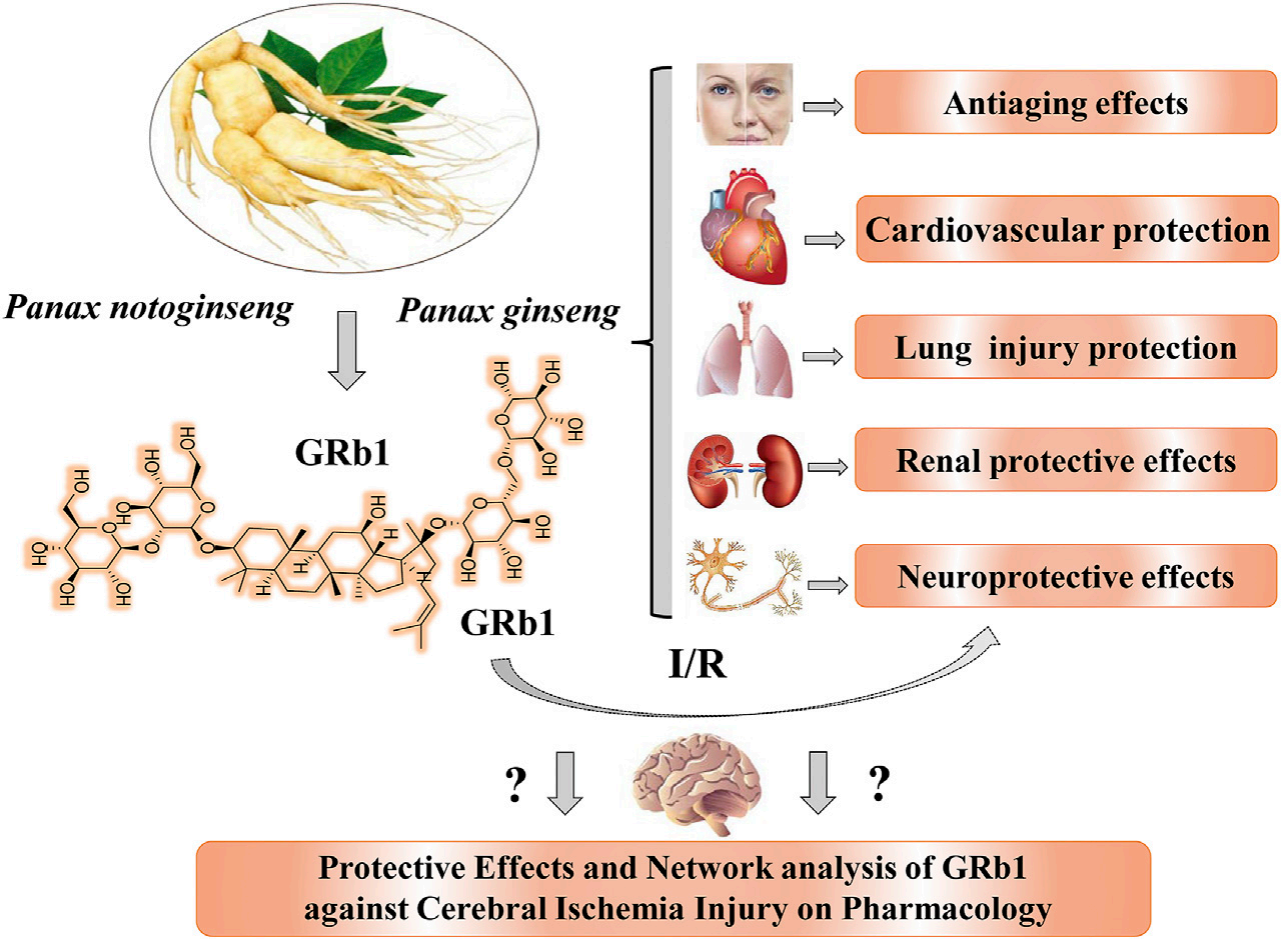
Natural sources, chemical structure, and main pharmacological activities of ginsenoside Rb1 obtained from *P. notoginseng* and *P. ginseng*. GRb1 exerts significant neuroprotective effects, but its efficiency and action network must be further explored and analyzed. GRb1, ginsenoside Rb1; I/R, indicates ischemia and reperfusion.

Ginseng Rb1 (GRb1) is a ginsenoside glycol (Liu et al., 2015; Ju et al., 2019) and one of the main active ingredients of *P. notoginseng* and *P. ginseng* (Figure 1). GRb1 has been proven to exert significant protective effects on the CNS, cardiovascular system (Xie et al., 2018a; Xie et al., 2018b), and immune system and possesses antitumor activities (Zhou et al., 2018; Zhou et al., 2019a; Zhou et al., 2019b). As shown in Figure 1, GRb1 possesses various pharmacological activities, including neuroprotective (Yang et al., 2008; Liu et al., 2013; Huang et al., 2015; Huang et al., 2015; Ye et al., 2019), acute renal injury-protective (Wang et al., 2008; Sun et al., 2012; Chen et al., 2019), cardiovascular-protective (Wang et al., 2008; Xia et al., 2011; Zheng et al., 2017; Li et al., 2020), lung injury-protective (Wang et al., 2013; Chen et al., 2014; Jiang et al., 2015; Li et al., 2015), and antiaging (Dong et al., 2017; Zhou et al., 2019a) effects, in many *in vitro* and *in vivo* models.

Currently, accumulated experiments and data suggest that GRb1 exerts neuroprotective effects both *in vivo* and *in vitro* and has a great potential as a novel candidate agent for ischemic stroke. To date, researchers have not clearly determined whether GRb1 can be used to treat ischemic stroke and cerebral I/R injury. No systematic review or analysis has been conducted to assess the protective effects and mechanisms by which GRb1 combats ischemic stroke and I/R injury (Figure 1). Hence, we conducted this analysis of preclinical studies of the effect of GRb1 on ischemic stroke. We searched the PubMed and China National Knowledge Infrastructure databases *via* using “Ginsenoside Rb1” and “Ischemia” as search terms. The PubMed database was comprehensively searched up to September 2020, and it showed 66 literatures; we excluded some irrelevant ones and then divided them into several aspects to further analyze the pharmacological effects and mechanisms of GRb1 in pre-treating and treating CIRI. Furthermore, we manually searched for other potential and relevant references, and there were no limitations in the language of all publications.

In this review, we found that GRb1 might alleviate cerebral/neural ischemia injury *via* its antiapoptotic, antioxidant, and anti-inflammatory activities, and effects on mitochondrial homeostasis, promoting neurogenesis, and improving brain functional connections and interactions, which provides more evidence for basic studies and further promotes the development of GRb1 as a candidate drug for the clinical treatment of ischemic stroke.

## Antiapoptotic Effects and Neurons

Recent reports have suggested that GRb1, a natural saponin ingredient of TCM, exerts remarkable neuroprotective effects on the CNS (Ahmed et al., 2016), prevents and alleviates cerebral I/R injury *via* anti-inflammatory activity, antioxidant activity, enhanced neuroproliferation and neurodifferentiation, and improved energy metabolism (Chen et al., 2010; Zhu et al., 2012; Huang et al., 2015), confirming that Rb1 exerts antiapoptotic effects on neurons.

First, *in vivo* experiments showed that Rb1 significantly inhibited CA1 neuronal death caused by a 2-vessel occlusion (2-VO) model in rats (Luo et al., 2014) and delayed neuronal death in gerbils (Lim et al., 1997); noticeably reduced the infarct size and neuronal deficits, relieved pathological changes (Yuan et al., 2007), and decreased the number of neural apoptotic cells (Wen et al., 1996) in rats with middle cerebral artery occlusion (MCAO) (Lim et al., 1997; Yuan et al., 2007; Yang et al., 2008); and scavenged free radicals (Lim et al., 1997) and improved hippocampal blood flow at 5 min after transient forebrain ischemia (Lim et al., 1997). Rb1 significantly prolonged the response latency of ischemic gerbils and rescued a significant number of ischemic CA1 pyramidal neurons (Wen et al., 1996; Lim et al., 1997). Under abnormal ischemic microenvironmental conditions, Rb1 evidently decreased the concentrations of glutamic acid and Ca^2+^ (Wang et al., 2017), noticeably alleviated memory deficits in rats, and reduced pyramidal cellular necrosis and apoptosis in the hippocampus induced by glutamate (Glu) and Ca^2+^ (Wang et al., 2017; Guo et al., 2018). Additionally, Rb1 suppressed the loss of BBB integrity by suppressing the induction of neuroinflammation in a model of ischemic stroke (Chen et al., 2015). Based on these results, Rb1, an effective neuroprotective drug, plays key roles in cerebral I/R injury.

Second, *in vitro* experiments (Table 1) suggested that Rb1 might increase cell viability, inhibit oxygen and glucose deprivation (OGD)–induced neuronal death, and reduce autophagic vacuoles in SH-SY5Y cells (Luo et al., 2014), changes that were blocked by the inhibitor LY294002. GRb1 significantly decreased the levels of free radicals, protected hippocampal neurons from lethal damage caused by the hydroxyl radical–promoting agent FeSO_4_ *in vitro* (Lim et al., 1997), and markedly suppressed the uptake of Glu and overload of Ca^2+^ in OGD-induced SH-SY5Y cells (Wang et al., 2017; Guo et al., 2018). GRb1 also significantly reduced the levels of lactate dehydrogenase (LDH) (Park et al., 2005; Li et al., 2016), nitric oxide (NO), and superoxide (O-) (Zhu et al., 2012; Huang et al., 2014). Thus, Rb1 might be regarded as an antiapoptotic agent with neuroprotective effects.

**TABLE 1:** Antiapoptotic effects of GRb1 on cerebral I/R injury based on current reports and results.

Type	Animal and dose	Model	Effect	Mechanism	CF
TGI	SD rat: 20–40 mg/kg SH-SY5Y: 1–10 μM	2-VO model OGD/R	↑ Cellular viability	↓ LC3II and Beclin1	Luo et al. (2014)
			↓ Neuronal death	↑ PI3K/phosphor-Akt	
			↓ Autophagic vacuoles	LY294002 Verify	
TCI	SD rats: 40 mg/kg	MCAO	↓ Pathological changes	↑ Bcl-2	Yang et al. (2008)
			↓ Apoptotic neural cells	↓ Bax	
TFI	Gerbils: 0.09–90 fM Hippocampal neurons	MCAO FeSO4 treatment	↓ Free radicals	↓ Oxidative damage	Lim et al. (1997)
			↑ response latency	↓ Apoptosis	
			↑ Hippocampal CA1 neurons		
TCI	SD rats: 40 mg/kg	MCAO	↓ Infarct and neuronal deficit	↑ GDNF	Yuan et al. (2007)
			↓ Apoptotic cells	↑ Bcl-2	
TCI	Gerbils: 10–20 mg/kg	MCAO	↑ response latency and synapses	↓ Apoptosis	Wen et al. (1996)
			↓ Pyramidal neurons		
TCI	SD rats: 40 mg/kg	MCAO	↑ Neurological functions	↑ BDNF	Gao et al. (2010)
			↑ Nestin-positive cells	↓ Caspase-3	
TGI	ICR mice: 5–40 mg/kg	MCAO	↓ Infarction and brain edema	↑ Arginase 1 and IL-10	Chen et al. (2015)
			↓ EB extravasation	↓ NOX-4 and NOX	
			↑ BBB integrity	↓ Free radicals	
			↓ MMP-9, IL-1β, and NO synthase	Neuroinflammation	
IAM	SD rats: 25–100 mg/kg SH-SY5Y cells: 10 μM	OGD/R Microperfusion	↓ Memory deficit pyramidal	↑ P-Akt/P-mTOR	Guo et al. (2018)
			↓ Necrosis and apoptosis	↓ P-PTEN	
			↓ Glu and Ca^2+^	Akt/mTOR/PTEN	
IAM	SD rats: 40 mg/kg	Microperfusion	↑ rCBF and GLT-1	↓ NMDAR and Cyt-C	Wang et al. (2017)
			↑ Neuronal ultrastructure	↓ Neuronal mitochondrial damage	
			↓ Glu and overload of Ca^2+^		

TGI, transient global ischemia; 2-VO, 2-vessel occlusion model; TCI, transient cerebral ischemia; TFI, transient forebrain ischemia; IAM, ischemic abnormal microenvironment; MCAO, middle cerebral artery occlusion; OGD/R, oxygen–glucose deprivation/reperfusion; GDNF, glial-derived neurotrophic factor; NO, nitric oxide; Glu, glutamate; MMP-9, matrix metalloprotein 9; IL, interleukin; BBB, blood–brain barrier; BDNF, brain-derived neurotrophic factor; rCBF, regional cerebral blood flow; GLT-1, glial glutamate transporter1; Cyt-C, cytochrome C; SD, Sprague–Dawley; NOX, NADPH oxidase; EB, Evans blue; PI3K, phosphoinositide 3-kinase; AKT, protein kinase B; NMDAR, N-methyl-D-aspartate-receptor; CF, cited references.

Furthermore, GRb1 significantly inhibited the expression of the proapoptotic genes Bax, Bad, and caspase-3 (Yang et al., 2008; Gao et al., 2010), upregulated Bcl-2 and the ratio of Bcl-2/Bax *in vivo* and *in vitro* (Yuan et al., 2007; Yang et al., 2008), increased the expression of glial-derived neurotrophic factor (GDNF) and brain-derived neurotrophic factor (BDNF) (Yuan et al., 2007; Gao et al., 2010), and thus prevented neuronal death induced by cerebral ischemia. GRb1 significantly increased the levels of Akt phosphorylated at Ser473 (P-Akt) and reduced the expression levels of LC3II and Beclin1, indicating that GRb1 might prevent ischemic neuronal death by modulating autophagy activation (Luo et al., 2014); moreover, GRb1 increased the levels of P-Akt and P-mTOR, reduced P-PTEN levels *in vivo* and *in vitro*, and ameliorated the abnormal microenvironment by activating the P-AKT/P-mTOR pathway and inhibiting P-PTEN (Guo et al., 2018). In contrast, the LY294002 treatment reversed these changes induced by GRb1. Furthermore, GRb1 inhibited the expression of NMDAR, increased the expression of glial glutamate transporter 1 (GLT-1), and downregulated the levels of cytochrome C (Cyt-C) in response to neuronal mitochondrial stress, which reduced the excessive Glu and Ca^2+^ levels (Wang et al., 2017; Guo et al., 2018).

In general, evidence from recent studies suggests that GRb1 may exert its antiapoptotic effects by regulating the phosphoinositide 3-kinase (PI3K)-protein kinase B (AKT)-TOR signaling pathways, inhibiting autophagy, alleviating mitochondrial stress and apoptosis pathways, modulating the expression of N-methyl-D-aspartate-receptor (NMDAR) and GLT-1, and improving neurogenesis and BDNF levels. Nonetheless, currently, the mechanisms by which GRb1 regulates ischemic neuronal apoptosis are not completely elaborated and summarized and should be further explored in the future.

## Regulation of Neuroinflammation and Microglia

Although CIRI is a complex pathology caused by the interaction of numerous pathophysiological factors (Allison and Ditor, 2014; Anderson et al., 2014), accumulating evidence indicates that acute inflammation and subsequent apoptosis and necrosis are involved in the progression of a cerebral ischemic insult (Jianhua et al., 2008; Eltzschig and Eckle, 2011; Jiang et al., 2014; Tao et al., 2015). Recently, GRb1 was reported to exert beneficial effects on cerebral ischemic stroke and to inhibit inflammatory cascades in the acute phases of cerebral ischemia (Wang et al., 2008; Zhu et al., 2012; Jiang et al., 2015).

According to recent studies (Table 2), GRb1 noticeably reduces the infarct volume, significantly alleviates neurological deficits, decreases neurological severity scores (Zhu et al., 2012; Liu et al., 2013; Liu et al., 2018; Chen et al., 2020), preserves the neuronal morphology and structure (Ke et al., 2014), improves pathological changes (Liu et al., 2018), and inhibits the activation of microglia in MCAO/R model rats (Zhu et al., 2012) and N9 microglia *in vitro* (Ke et al., 2014). These neuroprotective effects may be involved in microglia-mediated CNS inflammation and related neuronal damage in the acute phases of cerebral ischemia/hypoxia.

**TABLE 2:** Neuroprotective effects of GRb1 on cerebral ischemia injury are mediated by suppressing neuroinflammation and microglia-mediated inflammatory reactions, based on current reports and results.

Type	Animal and dose	Model	Effect	Mechanism	CF
*In vivo* TCI	SD rats: 40 mg/kg	MCAO	↓ TNF-α, IL-6	↓ p-NF-κB/p65	Zhu et al. (2012)
			↓ Activation of microglia	↓ NF-κB pathway	
*In vitro*	Cortical neurons N9 microglia	Hypoxic co-culture	↑ Cell viability	↓ Neuronal apoptosis	Ke et al. (2014)
			↑ Neuronal morphology	↓ Caspase-3 and microglia	
			↓ NO, superoxide, and TNF-α	↓ Inflammatory reaction	
*In vivo* TCI	SD rats: 40 mg/kg	MCAO	↓ Neurologic defect degree	↓ IL-1β	Liu et al. (2013)
			↓ Cerebral infarction volume	↓ Inflammatory damage	
*In vivo* TCI	SD rats: 50–100 mg/kg	MCAO	↓ TNF-α and IL-6	↓ NF-κB pathway	Liu et al. (2018)
			↓ Infarct volume	↓ Cleaved caspase-3	
			↓ Neuronal apoptosis	↓ Caspase-9 and HMGB1	
			↓ NO synthase and NO		
*In vivo* TGI	ICR mice: 5–40 mg/kg	MCAO	↑ BBB integrity	↓ Free radicals	Chen et al. (2015)
			↓ EB extravasation	↑ Arginase 1 and IL-10	
			↓ Infarction and brain edema	↓ NOX-4 and NOX	
			↓ MMP-9, IL-1β, and NO synthase	↓ Neuroinflammation	
*In vivo* TGI	SD rats: 50 mg/kg Probiotic	Pseudo germ-free, MCAO	↓ Infarct size	↑ GABA	Chen et al. (2020)
			↓ Neurological deficit score	↑ Probiotics *Lac.H*	
			↓ IL-1β, IL-6, and TNF-α	↑ GABA receptors	

TGI, transient global ischemia; TCI, transient cerebral ischemia; IL, interleukin; SD, Sprague–Dawley; MCAO, middle cerebral artery occlusion; TNF-α, tumor necrosis factor α; NF-κB, nuclear factor-κB; NO, nitric oxide; MMP-9, matrix metalloprotein 9; BBB, blood–brain barrier; NOX, NADPH oxidase; EB, Evans blue; HMGB1, high mobility group protein-1; CF, cited references.

On the one hand, ginsenoside GRb1 notably decreased the activation of microglia induced by I/R (Zhu et al., 2012) and a hypoxic coculture system (Ke et al., 2014) and significantly reduced the levels of the interleukin (IL)-1β (Zhu et al., 2012; Liu et al., 2013; Chen et al., 2015; Chen et al., 2020), tumor necrosis factor-α (TNF-α) (Zhu et al., 2012; Ke et al., 2014; Liu et al., 2018; Chen et al., 2020), IL-6 (Zhu et al., 2012; Liu et al., 2018; Chen et al., 2020), and high mobility group protein 1 (HMGB1) mRNAs and proteins in brain tissue and serum (Liu et al., 2018); on the other hand, GRb1 significantly decreased the levels of NO (Chen et al., 2015), superoxide (Ke et al., 2014; Liu et al., 2018), and nitric oxide synthase (Liu et al., 2018) produced by microglia *in vitro* (Ke et al., 2014) and *in vivo* (Zhu et al., 2012; Liu et al., 2018), reduced neuronal apoptosis (Ke et al., 2014; Liu et al., 2018), and reduced the levels of cleaved caspase-3 and caspase-9 (Ke et al., 2014; Liu et al., 2018).

Further studies revealed that GRb1 inhibited nuclear factor kappa B (NF-κB) signaling pathways by decreasing the levels of phosphorylated NF-κB/p65 and IB-kinase complex (IKK) and downregulating HMGB1 and its related local inflammation (Ke et al., 2014; Liu et al., 2018), which stimulate NF-κB translocation and mitogen-activated protein kinase (MAPK) phosphorylation triggered by HMGB-1/TLR4 (Lu et al., 2011; Procaccio et al., 2014; Sun and Nan, 2016; Xie et al., 2019). Moreover, GRb1 reduced the expression levels of proinflammatory factors, attenuated the activity of MMP-9 (Chen et al., 2015), upregulated the expression of γ-aminobutyric acid (GABA) receptors in I/R rats (Chen et al., 2020), protected BBB integrity in ischemic stroke by suppressing neuroinflammation, decreased the production of MMP-9 and NOX4-derived free radicals (Chen et al., 2015), and regulated the probiotic *Lac.H* and GABA receptor levels (Chen et al., 2020). Overall, GRb1 may exert a neuroprotective effect on I/R-induced injury by suppressing neuroinflammation and microglia-mediated inflammatory reactions.

## Antioxidant Effects and Mitochondria

Oxidative stress plays an important role, and reactive oxygen species (ROS) is implicated in the tissue damage that occurs in cerebral ischemic pathogenesis, which results in the production of toxic molecules that alter cellular proteins, lipids, and ribonucleic acids, leading to cell dysfunction or death (Crack and Taylor, 2005; Chamorro et al., 2016). Excessive ROS severely impair mitochondria and their related energetic metabolism functions. GRb1 exhibits various pharmacological activities, including antioxidant (Dong et al., 2017; Li et al., 2019; Xu et al., 2019; Ye et al., 2019), antiapoptotic, and neuroprotective properties.

First, the results of *in vivo* experiments (Table 3) showed that GRb1 protected hippocampal CA1 neurons (Lim et al., 1997), reduced free radicals (Chen et al., 2015), and possessed potential for treating brain injuries (Yang et al., 2008); meanwhile, GRb1 increased superoxide dismutase (SOD) activity, decreased malondialdehyde (MDA) contents in the serum and spinal cord tissue (Ye et al., 2019), and inhibited oxidative stress and extracellular signal-regulated kinase (ERK) activation in aged mice exposed to cerebral ischemia (Dong et al., 2017). All of the antioxidant effects of GRb1 are beneficial for reducing neuronal death, mitochondrial damage, and astrocyte injury induced by I/R, indicating that GRb1 may reduce pathological changes and decrease neural cell apoptosis (Yang et al., 2008; Chen et al., 2015).

**TABLE 3:** Antioxidant effects of GRb1 on I/R neuronal injury mediated by the inhibition of oxidative stress and mitochondrial injury, increases in energy metabolism and protection of the BBB, based on recent reports and results.

Type	Animal and dose	Model	Effect		Mechanism	CF
*In vitro* I/R	SD rats Astrocyte: 6.71–32.00 mg/ml	OGD/R		↓ Spinal cord edema	↑ AQP	Li et al. (2019)
				↑ Neurological function	↑ NGF	
				↓ Cellular membrane permeability	↑ BDNF	
*In vivo* TCI	SD rats: 40 mg/kg	MCAO		↑ Nestin-positive cells	↑ BDNF	Gao et al. (2010)
				↑ Neurological functions	↓ Caspase-3	
				Histological feature	↑ Promotion of neurogenesis	
*In vitro* I/R	C57BL/6J Mice Astrocytes: 10 μM	OGD/R		↓ Intracellular ROS	↑ Efficiency of mitochondrial oxidative phosphorylation	Xu et al. (2019)
				↑ Cell viability, CAT, and ATP		
				↑ mtDNA copy number and MMP		
				↑ Complexes I, II, III, and V		
*In vivo* TCI	C57 Mice: 0.5–10 mg/kg	MCAO		↓ Brain trauma	↓ ERK activation	Dong et al. (2017)
				↓ NOX-1, -2, and -4	↓ Oxidative stress	
				↓ NADPH oxidase gen		
*In vivo* TGI	ICR mice: 5–40 mg/kg	MCAO		↑ BBB integrity	↓ Free radicals	Chen et al. (2015)
				↓ EB extravasation	↑ Arginase 1 and IL-10	
				↓ Infarction and brain edema	↓ NOX-4 and NOX	
				↓ MMP-9, IL-1β, and NO synthase	↓ Neuroinflammation	
*In vivo* SCII	SD rats: 20–80 mg/kg	SCII		↑ SOD; ↓ MDA	↓ Apoptosis	Ye et al. (2019)
				↑ Neurological function	↑ Survivin protein	
*In vivo* SCII	SD rats: 15 mg/kg	SCII		↓ Spinal cord apoptosis	↑ Bcl-2/Bax ratio	Zhao et al. (2018)
				↑ Hindlimb locomotor function	↑ Caspase-3 and p-Ask-1	
*In vivo* TCHI	Mongolian gerbils: 250 μg/ml	Occluding BVA		↓ Hearing loss	↓ Neural cell apoptosis	Fujita et al. (2007)
				↓ Auditory brainstem response		

SCII, spinal cord I/R injury; TGI, transient global ischemia; TCI, transient cerebral ischemia; TCHI, transient cochlear ischemia; SD, Sprague–Dawley; NO, nitric oxide; MMP-9, matrix metalloprotein 9; BBB, blood–brain barrier; NOX, NADPH oxidase; EB, Evans blue; OGD/R, oxygen–glucose deprivation/reperfusion; MCAO, middle cerebral artery occlusion; AQP, aquaporin; NGF, nerve growth factor; BNDF, brain-derived neurotrophic factor; ROS, reactive oxygen species; CAT, catalase; ATP, adenosine triphosphate; MMP, mitochondrial membrane potential; mtDNA, mitochondrial DNA; ERK, extracellular signal-regulated kinase; SOD, superoxide dismutase; MDA, malondialdehyde; BVA, bilateral vertebral arteries; CF, cited references.

Second, *in vitro* experiments (Table 3) confirmed that GRb1 significantly improved cell viability, decreased intracellular ROS production, and increased catalase (CAT) activity and the mtDNA copy number in OGD/R-induced astrocytes, thus inhibiting oxidative phosphorylation (OXPHOS) (Xu et al., 2019). GRb1 exerted obvious protective effects on the mitochondria by distinctly attenuating MMP depolarization, improving the efficiency of mitochondrial OXPHOS, increasing the activities of complexes I, II, III, and V, and increasing the level of adenosine triphosphate (ATP) following OGD/R induction (Xu et al., 2019).

Further studies (Table 3) revealed the antioxidant effects of GRb1. On the one hand, GRb1 significantly downregulated NOX-4 expression and NOX activities in ischemic rat brain tissues (Chen et al., 2015; Dong et al., 2017), inhibited ischemia-stimulated NADPH oxidase gene expression, including NOX-1, 2, and 4 (Dong et al., 2017), and prevented ERK activation in aged mice (Dong et al., 2017). On the other hand, GRb1 significantly suppressed mitochondrial damage, increased the mitochondrial membrane potential (MMP) in OGD/R-induced astrocytes (Xu et al., 2019), and thus improved the antioxidant activity (CAT and SOD). GRb1 remarkably increased aquaporin (AQP) 4 levels (Li et al., 2019), decreased Bax expression (Yang et al., 2008), and upregulated Bcl-2 (Yang et al., 2008), nerve growth factor (NGF), and BDNT expression (Li et al., 2019) *in vivo* and *in vitro*. Thus, GRb1 is potentially useful for treating brain injury, due to its antioxidant effects and mitochondrial protection.

## Inhibition of Other Types of Neural Ischemic Injury

Related studies have shown that GRb1 significantly alleviates I/R injury of the kidney (Wang et al., 2008; Sun et al., 2012; Sun et al., 2013; Chen et al., 2019) and brain (Chen et al., 2010; Zhu et al., 2012; Huang et al., 2015). However, few reports have assessed spinal cord ischemia–reperfusion injury (SCII). According to recent studies, GRb1 reduces cell apoptosis induced by SCII by inhibiting oxidative stress (Ye et al., 2019). GRb1 noticeably improves hindlimb locomotor dysfunction in rats (Zhao et al., 2018), increases SOD activity, decreases the MDA content in serum and spinal cord tissue (Ye et al., 2019), and inhibits neuronal apoptosis. The potential mechanisms may be tightly associated with promoting the expression of survivin (Ye et al., 2019), downregulating the levels of caspase-3 and phosphorylated Ask-1 (p-Ask-1), and improving the Bcl-2/Bax ratio (Zhao et al., 2018) in SCII rats. After the exposure of spiral ganglion cells (SGCs)to transient cochlear ischemia, GRb1 significantly reduced the percentage of the auditory brainstem response threshold shift and prevented hearing loss caused by ischemic injury to SGCs in Mongolian gerbils (Fujita et al., 2007), indicating that GRb1 might protect SGCs from ischemic injury.

In addition, GRb1 clearly increased the expression of NGF in OGD/R-stimulated astrocytes (Li et al., 2019), upregulated BDNF expression, and downregulated caspase-3 expression in MCAO/R model rats (Yuan et al., 2007; Gao et al., 2010). Therefore, GRb1 may exert neuroprotective effects by promoting neurogenesis.

## Analysis of Safety and Therapeutic Effectiveness

Based on the above summarized findings (Tables 1–3), Rb1 has a potential therapeutic value for ischemic stroke *via* immunological, antioxidant, and neuroprotective effects. However, the efficacy and safety of Rb1 for ischemic stroke in human have not been tested, and its effective dose is not known. Nevertheless, there is evidence that red ginseng extract, which contains 20–30% of GRb1, at 23, 25, 50, and 100 mg with repeated oral administration for 1–2 weeks, is safe and well tolerant in healthy subjects (Kim, 2013; Jin et al., 2019; Choi et al., 2020). In addition, randomized controlled studies showed that 200–400 mg of ginsenosides or its preparations significantly ameliorated neurological deficit (He et al., 2011) and improved working memory and daily activities of patients with ischemic stroke (Reay et al., 2010; He et al., 2011), indicating that a daily dose of 40–120 mg of GRb1 may be safe and effective for treating ischemic stroke. The effective dose of Rb1 tested in animal studies with notably attenuated ischemia-induced cerebral I/R injury ranges from 10 to 40 mg/kg (Tables 1–3), which is equivalent to 0.8–3.2 mg/kg in human (calculated from dose in mice) or 48–192 mg Rb1 for a 60 kg person. Thus, an effective concentration of Rb1 may be achievable in human.

## Conclusion and Remarks

**FIGURE 2:**
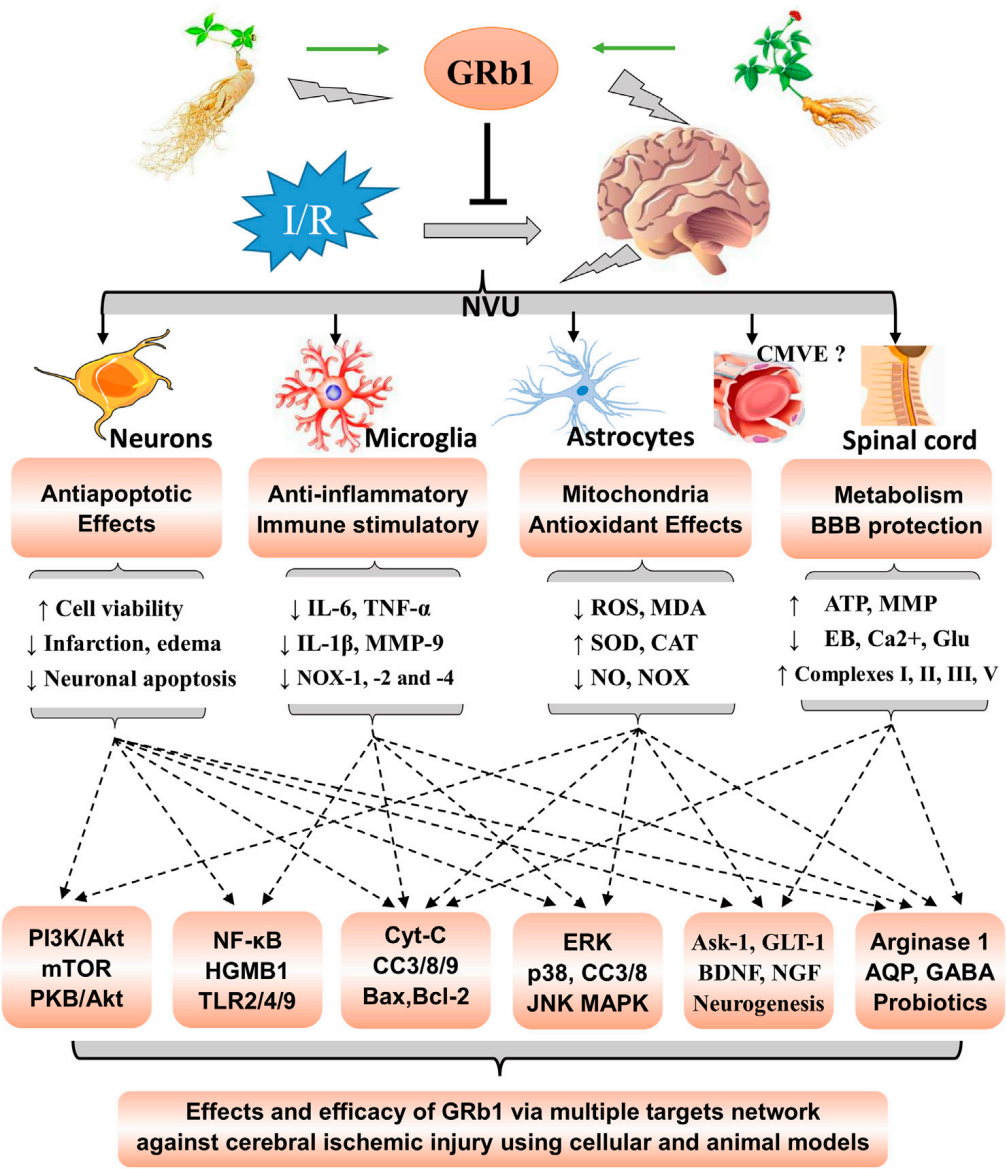
Summarized effects and molecular network analysis of GRb1 in cerebral ischemic injury. Rb1 exerts significant neuroprotective effects on the neurovascular unit (NVU) and other neural cells *via* the network actions of its antiapoptotic, antioxidant, and anti-inflammatory activities; mitochondrial homeostasis; neurogenesis promotion; and regulation of the probiotic balance. The molecular mechanisms involve multiple effects, multiple targets, and multiple pathways. NVU, neurovascular unit; CMVE, cerebral microvascular endothelium. GRb1, ginsenoside Rb1; I/R, indicates ischemia and reperfusion. CC, cleaved caspase.

GRb1, a natural active ingredient, exerts neurotrophic and neuroprotective effects on the CNS. In the present review (Figure 2), we summarized the available reports on the therapeutic effects and the molecular mechanisms of GRb1 in cerebral I/R injury. The currently available data and our summarized results suggest that GRb1 may alleviate cerebral/neural ischemic injury *via* multiple pharmacological activities, such as its antiapoptotic, antioxidant, and anti-inflammatory properties, preservation of mitochondrial homeostasis, promotion of neurogenesis, and maintenance of the probiotic balance. Based on these results, GRb1, a special natural compound, has bright prospects for the prevention and treatment for ischemic stroke with a pharmacological network of multiple effects, targets, and molecular pathways.

Cerebral I/R injury is a complicated pathological process (Turley et al., 2005; Eltzschig and Eckle, 2011; Chamorro et al., 2016) that includes various types of I/R damage to the neurovascular unit (NVU), which is composed of nerve cells, BBB, microglia, and extracellular matrix (Lok et al., 2007; Guo and Lo, 2009; Lo, 2017). For many decades, neuronal injury has been considered the main cause of functional deficits after brain injury. Accordingly, almost all therapeutic strategies were targeted at rescuing neurons and repairing neuronal damage. However, emerging data from both experimental models and patients now clearly show that, in patients with stroke, saving neurons alone may also be insufficient for treating brain infarcts (Lok et al., 2015) as brain functional connections and interactions among the different components in the NVU are also important (Cai et al., 2017; Jiang et al., 2018). Coincidentally, as shown in Figure 2, GRb1 exerts significant antiapoptotic effects on neurons exposed to cerebral ischemia stroke, suppresses neuroinflammation and microglia-mediated inflammatory stress in the acute phase of I/R, and protects ischemia-exposed astrocyte cells. Meanwhile, GRb1 inhibits oxidative stress and mitochondrial damage in cells exposed to I/R, preserves the BBB integrity, improves energy metabolism, and promotes neurogenesis. Moreover, GRb1 may alleviate spinal cord ischemia–reperfusion injury (SCII) and regulate the probiotic balance (Figure 2). Hence, GRb1 obviously plays a vital role not only in neural protection but also *via* the inhibition of NVU damage caused by CIRI.

From the perspective of protecting the NVU, GRb1 prevents I/R-induced cell apoptosis and death, suppresses excessive Ca^2+^, glutamate, and RNS levels, alleviates mitochondrial stress, improves neurogenesis, and reduces the cerebral infarct volume *in vitro* and *in vivo*, indicating that GRb1 may exert antiapoptotic effects and protect the NVU from CIRI (Figure 2). Although the mechanisms have not been completely elaborated, our summarized results (Table 1) further reveal the molecular mechanisms and vital biomarkers of the effects of GRb1 on CIRI, which may be related to the PI3K-AKT-mTOR, autophagy, and mitochondrial apoptosis signaling pathways, NMDA receptor and GLT-1 targets, and neurogenesis.

Neuroinflammation underlies the etiology of multiple neurodegenerative diseases and stroke (Eldahshan et al., 2019). In the acute phase of ischemic stroke, resident microglia and recruited macrophages assume an M2 phenotype, and the immune-mediated inflammatory response is activated, resulting in the secretion of a number of inflammatory cytokines (IL-6, IL-1β, and TNF-α and the proteolytic enzymes MMP 3 and 9) (Jiang et al., 2018). Proinflammatory cytokines further trigger severe inflammatory stress, aggravate BBB dysfunction (Jiang et al., 2018), and induce NVU damage. All these events lead to the exacerbation of ischemic injury. Based on the summarized results listed in Table 2, we found that GRb1 inhibited the NF-KB, HMGB-1/TLR4, and HMGB1 signaling pathways, regulated the MAPK signaling pathway, increased the expression of GABA receptors, and reduced the levels of proinflammatory cytokines (IL-6, MMP-9, IL-1β, and TNF-α). By regulating these pathways, GRb1 suppresses neuroinflammation and microglia-mediated inflammatory reactions, reduces the damage caused by inflammatory factors, improves the integrity and normal functions of the BBB, and thus inhibits necrosis and apoptosis associated with anti-inflammatory activities at the early stages after stroke, which plays a fundamental role in the maintenance of CNS homeostasis, BBB structural integrity, and normal functions of the NVU.

The neutralization of oxidative and nitrosative stresses is a potential therapeutic strategy because the ischemic brain is highly susceptible to oxidative damage (Crack and Taylor, 2005; Xie et al., 2020). After CI/RI, the production of ROS increases, leading to lipid peroxidation, mitochondrial and DNA damage, protein nitration, and mitochondrial injury that evokes the mitochondrial release of apoptosis inducers (Bolaños et al., 2009; Chamorro et al., 2016). According to recent studies (Table 3), GRb1 decreases the production of ROS, MDA, and NO, improves antioxidant defenses (CAT, SOD, and GSH-px), and inhibits I/R-induced mitochondrial injury *in vivo* and *in vitro*. Moreover, it remarkably increases AQP4 levels (Li et al., 2019), decreases Bax expression (Yang et al., 2008), upregulates the expression of Bcl-2 (Yang et al., 2008), downregulates NOX-4 expression and NOX activities, and inhibits the cascade reactions of caspase-3 and caspase-9. Thus, GRb1 exerts significant neuroprotective effects due to its antioxidant activity and protects the mitochondria in the treatment of cerebral ischemic injury.

In summary, this review addresses the effects and efficacy of GRb1 by discussing research results obtained using cellular and animal models; the summarized results and analysis indicate that GRb1, a new candidate agent, has bright prospects for preventing and treating ischemic stroke.

However, unrecognized actions and limitations of GRb1 still exist. 1) Researchers have not clearly determined whether GRb1 alleviates ischemic injury of the cerebral microvascular endothelium (CMVE) of the NVU. 2) Because of the lack of clinical testing and validation of preclinical data, the use of GRb1 as a new drug for treating ischemic stroke remains a challenge. 3) The existing research and data only showed the effects of GRb1 on I/R in the early stages and acute phases. Therefore, currently available clinical trials and data collection are urgently needed, and future studies should focus on the effects and molecular mechanisms of GRb1 on the NVU system and the recovery phase of ischemic stroke.
